# Neural Signatures of the Reading-Writing Connection: Greater Involvement of Writing in Chinese Reading than English Reading

**DOI:** 10.1371/journal.pone.0168414

**Published:** 2016-12-16

**Authors:** Fan Cao, Charles A. Perfetti

**Affiliations:** 1 Department of Communicative Sciences and Disorders, Michigan State University, East Lansing, MI, United States of America; 2 Learning Research and Development Center, University of Pittsburgh, Pittsburgh, PA, United States of America; University of Akron, UNITED STATES

## Abstract

Research on cross-linguistic comparisons of the neural correlates of reading has consistently found that the left middle frontal gyrus (MFG) is more involved in Chinese than in English. However, there is a lack of consensus on the interpretation of the language difference. Because this region has been found to be involved in writing, we hypothesize that reading Chinese characters involves this writing region to a greater degree because Chinese speakers learn to read by repeatedly writing the characters. To test this hypothesis, we recruited English L1 learners of Chinese, who performed a reading task and a writing task in each language. The English L1 sample had learned some Chinese characters through character-writing and others through phonological learning, allowing a test of writing-on-reading effect. We found that the left MFG was more activated in Chinese than English regardless of task, and more activated in writing than in reading regardless of language. Furthermore, we found that this region was more activated for reading Chinese characters learned by character-writing than those learned by phonological learning. A major conclusion is that writing regions are also activated in reading, and that this reading-writing connection is modulated by the learning experience. We replicated the main findings in a group of native Chinese speakers, which excluded the possibility that the language differences observed in the English L1 participants were due to different language proficiency level.

## Introduction

How the reading brain accommodates the variety of languages and writing systems is an interesting question, given the relatively recent addition of literacy as a human skill, which sets it outside the more universal neural bases of sensory, perceptual, and language systems. To the extent that all reading depends on the connection of visual input with language areas, some universality is to be expected [1] and indeed has been found in the intersection of reading and language areas in cross-language imaging research [2,3]. This intersection of spoken and written language areas recently has been confirmed across four-languages, including Chinese [4]. However, within the larger picture of universality, variations due to language and writing system are expected and these variations also have been reported. For example, reading in a more transparent language such as Italian or Spanish is associated with greater activation in the temporo-parietal regions involved in grapheme-phoneme-conversion or assembled phonology, whereas reading in English, a deep orthography, is associated with greater activation in a ventral reading pathway (including the inferior temporal gyrus) that might support processes involved in semantically supported word retrieval [5,6].

Compared with observations within alphabetically written languages, a contrast between alphabetic and Chinese reading provides more compelling differences that arise from basic writing system design and from the forms and numbers of graphic units. Chinese-English comparisons have been found in visuo-orthographic regions (more involved in Chinese reading) and phonological regions (more involved in English reading) [7]. For example, bilateral superior parietal lobules, middle occipital gyri and fusiform gyri show more activation in Chinese reading, whereas left inferior frontal gyrus, superior temporal gyrus and inferior parietal lobule are more involved in English reading [3]. Another consistent finding across studies is a greater involvement of the left middle frontal gyrus (LMFG) in Chinese than in English, which has been established in previous studies including two meta-analysis studies [2,3,8,9]. This region is in the dorsal extent of Broca’s area, and the ventral part of premotor cortex, precentral gyrus, with convergent locations reported in meta-analyses, (-48,9,30) in Bolger et al (2005) [3] and (-46,18,28) in another meta-analysis study by Tan et al (2005) [2]. This region has been found to show a children-to-adult developmental increase for Chinese at (-46,8,34) in a visual rhyming and visual spelling task [10], a reading-skill-related increase in Chinese children during a visual rhyming judgment task at (-39,21,36) [11], and reduced activation in children with dyslexia in a homophone judgment task at (-50,11,34) [12], suggesting its critical role in Chinese reading.

However, there is not a consensus on the functional significance of this region. One suggestion is that the left middle frontal gyrus supports the whole-syllable (addressed phonology) procedure required in Chinese reading [2]; another, that it is related to the tonal nature of Chinese phonology [13]; a third, that it allows memory based lexical integration of orthography, phonology and semantics [14]; a fourth, that it supports the complex visual analysis of Chinese characters [15]. Even within the English literature, this region has been found to be involved in multiple functions such as lexical semantics [3]; phonological processing [16], lexical selection [17], grapheme–phonology conversion [18], lexical retrieval [19], and the orthographic lexicon [20].

On the other hand, this left MFG has been repeatedly found to be involved in writing—at (-50,6,26) in a meta-analysis study by Planton 2013 [21] and (-44,6,28) in another meta-analysis study by Purcell 2011 [22]. This region consistently activates in writing tasks, along with Exner’s area, which is more medial and dorsal. Exner’s area has long been considered important for the coding of grapheme-writing movements in memory [23]. Lesion studies suggest that impaired Exner’s area causes agraphia with damaged written production of graphemes but preserved orthographic representation [24,25]. Exner’s area is consistently found to be activated in writing-related hand motor planning with a center in BA 6 around (-24,-5, 51) and extending dorsally into the SFG/SFS (-26, -4, 62) [22,24]. In contrast, the role of the left MFG in writing has drawn much less attention and is not well understood, even though the two meta-analyses suggest that this region is involved in central-linguistic aspects of writing, observed in both reading and writing. In contrast, Exner’s area is associated with the peripheral processes of writing [21,22]. The left MFG has been found to be more involved in writing than in reading at (-48,6,28) [26], more involved in writing low frequency words than high frequency words at (-49,1,33) [27], selectively sensitive to forward handwriting gestures but not static words at (-42, 6, 20) [28], and more activated in the right homologue in left handed writers at (51, -2, 39) [29]. Given its location between Broca’s area and premotor cortex, the left MFG may have a role in the translation from linguistic representation to writing related motor information.

The LMFG that has been reported to be more involved in Chinese reading than English overlaps with the LMFG that has been reported to be involved in writing, with distance between the peaks as close as 3.6 mm [3,26]. With these considerations in mind, we can offer an explanation for why the left MFG is more involved in Chinese reading than in English reading. Our hypothesis is that the left MFG has a role in connecting a graphic representation to writing-specific motoric processes and that these processes are more involved as part of reading in Chinese than in English. This is because Chinese characters are learned through repeated copying and writing, during which, the character is written in a specific sequence of strokes. This extensive experience leads to an orthographic representation that includes a motor program for writing. Chinese children have to learn and remember the meaning and pronunciation of several thousand characters, and repeated writing of the characters supports this demanding task [30,31]. Consistent with this reading-writing connection, the ability to copy characters has significant power in predicting Chinese children’s reading performance even after phonological awareness is accounted for [32]. This contrasts with English, whose alphabetic system, even with its inconsistencies, places the instructional emphasis not on writing but on reading, usually with some attention to grapheme-phoneme connections and sight reading of higher frequency irregular words.

The hypothesis of writing-specific motor processes can be considered an example of the motor-percept common coding principle [33]. On this principle, percepts and actions are linked through a shared representation and thus are mutually activated during either perception or action. Applied to reading, this principle suggests that seeing a graphic form activates the action—the writing movements—associated with that form. Neural evidence for this motor-perception linkage is grounded in the discharge of ventral premotor neurons when, in the absence of actual movements, a monkey is merely looking at graspable objects [34]. In humans, this region has been found to activate during attention to manipulable objects such as tools [35–39]. In reading, imaging studies have found that viewing letters/words/characters that were learned through writing evoked greater activation in this region than viewing those learned by passive viewing. This effect was located in the left IFG at (-56, 4, 19) [40], at (-42, 6, 20) [28], the ventral premotor cortex at (-51,-2,41) [41], the ventral precentral gyrus at (-49,-5,44) [42] and (-53,-6,41) [43]. These regions are all proximal to the left MFG reported to be differentially active in Chinese versus English.

With the role of nearby pre-motor regions and Exner’s areas established for writing, we hypothesize that the LMFG serves as a linguistic-kinesthetic mediator, an orthographic memory of the written form that functions in its writing. To the extent that an individual’s history includes writing that word, its form will activate associated motoric components that are part of that history. Thus, our study aimed to test the hypothesis that the left MFG is more involved in Chinese reading than in English reading because a kinesthetic component of writing—a specific motor sequence of stroke production—is also part of the Chinese orthographic representation. This motorically-enriched orthographic representation affects the visual recognition of the characters through the left MFG, which links the visual form of the character to the motor movements that produce it.

To test this hypothesis, we re-analyzed data from a study of English learners of Chinese [8] with new comparisons between tasks and languages. English L1 participants engaged in both reading and writing tasks in both Chinese and English, allowing four conditions to be compared. We also replicated this design with a group of Chinese L1 participants to rule out the possibility that language effects observed for English L1 participants were due to L2 difficulty. We expected the left MFG to be more activated in writing than in reading regardless of language and more activated in Chinese than in English regardless of task. Furthermore, in order to directly test whether this region is associated specifically with writing-related motor information in the English L1 learners of Chinese [8], we manipulated how the Chinese characters were learned by the participants. Half of the characters were learned through character- writing and half were learned through pinyin-writing, an alphabetic system in which Roman alphabet letters correspond to Chinese phonemes, indicating the pronunciation of the character. Pinyin essentially served as a control for “mere writing”, allowing the inference that effects observed when a character was viewed were because the participant wrote that character during learning.

To place the present study in the context of Cao et al (2013), we emphasize the following: Cao et al (2013) reported that English learners of Chinese showed greater activation in bilateral superior parietal lobule, lingual gyri and sensori-motor cortex in both a lexical decision task and an imagined writing task when they viewed characters that they had learned through character-writing compared with pinyin-writing. The current study incorporates those data—English learners of Chinese responding to Chinese characters they had learned—into its design, but makes novel comparisons to the performance of these English L1 participants on English word reading and writing, data that were not reported in Cao et al (2013); a comparison of tasks: reading and imagined writing that was not made for the English L1 learners of Chinese in Cao et al, 2013; and a new sample of data on the same tasks collected from native Chinese speakers. Thus, the present study is a novel comparison of previously unreported data on reading (passive viewing) and imagined writing from the study of Cao et al (2013) with new results from a new sample of Chinese native speakers who are bilingual in English. Data from the Chinese native speakers can confirm that differences between Chinese and English reading and writing observed for English L1 speakers are due to the writing systems rather than to Chinese language proficiency.

## Method

### Participants

The sample of English learners of Chinese is fully described in Cao et al (2013). They were 17 undergraduate students who were native speakers of English enrolled in introductory Chinese at Carnegie Mellon University (CMU) or the University of Pittsburgh (UPitt). The new sample 17 bilingual native speakers of Chinese were also students, undergraduate or graduate, from CMU and UPitt. They included thirteen females, mean age = 24.9, age range = 19–29. (For comparison, the English L1 sample included seven females and had a mean age-21.58, range 19–24.) Based on an informal interview, all participants met the following criteria: (1) right-handed, (2) free of neurological disease or psychiatric disorders, (3) no Attention Deficit Hyperactivity-Hyperactivity Disorder (ADHD), and (4) no learning disability. The Institutional Review Boards at both the University of Pittsburgh and Michigan State University reviewed and approved this study and the consent procedure. Written consent forms were obtained from participants.

### Procedure

#### Behavioral training and testing

As reported in Cao et al (2013), the English L1 participants learned 30 Chinese characters in each of two training conditions (i.e. character writing and pinyin writing) for 5 consecutive days. The characters were selected from the participants’ Chinese textbook and had not been taught prior to the experiment. A pretest confirmed that the participants knew none of the 60 characters. In each training session, all 60 characters were taught. During a training trial, each character was first presented alone in the center of a computer screen for 800 ms; then the character’s pinyin spelling appeared and remained displayed for 800 ms with simultaneous presentation of the auditory recording of the character’s pronunciation, which had been produced by a native Chinese speaker; finally, an English translation of the character was presented, also for 800ms. This sequence was followed by a 15-s pause, while the participant was required to write from memory either the character or its pinyin using paper and pencil, depending on the training condition, three times. The entire sequence of character presentation plus writing prompt was repeated three times in a row for each character. On each day of training, a computerized test was conducted after the learning session using a character-pronunciation matching task and a character-meaning matching task. After learning and testing on the last day of training, participants completed a paper-pen post-test that assesses their proficiency on trained characters.

#### fMRI session

The fMRI session followed within one week after the last training day for the English L1 participants. Both a passive viewing task and an imagined writing task were conducted for all participants. A lexical decision task was also conducted as part of a bigger project, which was not the focus of the current study [8]. The passive viewing task, which requires minimal lexical processing, was presented first to avoid the influence of the imagined writing task, which engages more detailed lexical processing. A block design was used for the passive viewing task and an event-related design was used for the imagined writing tasks. There was a 12-second equilibration period at the beginning of each fMRI run and a 22-second period at the end to deconvolve the hemodynamic response function (HRF) for the last trial.

#### Passive viewing task

In this task, participants were instructed to view a stimulus presented at the center of the screen. Four types of stimuli were presented: 30 Chinese characters learned in the character-writing condition, 30 characters learned in the pinyin-writing condition, 30 English words, and fixation baseline blocks. The English words were matched with the English translation of the Chinese characters in frequency [44]. Six five-item blocks were presented for each stimulus type; each stimulus was presented for 800 ms followed by a 200 ms blank. The three experimental blocks were interleaved with baseline blocks, during which a fixation (+) was presented using the same procedure as the experimental stimuli. The passive viewing task lasted 3 minutes and 5 seconds.

#### Imagined writing task

In this task, participants were instructed to imagine writing with their index finger with the stimulus presented briefly on the screen. The imagined finger writing task was adopted from “mental imagery of writing” used in previous studies in Japanese writing [45] and English writing studies [46,47]. This task can limit head motion caused by actual handwriting tasks used in fMRI studies. Previous studies have compared mental imagery of motor processes vs. actual motor processes and found that they require a massive overlapping network in the primary motor and motor association cortices [48–51]. Therefore, our imagined writing task fulfilled our purpose to construct a comparison to the reading task by engaging the linguistic and motoric process of orthographic production. 60 learned Chinese characters and 30 English words were used as stimuli which were the same as in the passive viewing task. 45 null trials with a fixation (+) were added as a baseline, during which participants were asked to imagine writing the fixation (+) with their index finger. Each stimulus was presented for 200 ms followed by a blank of 1,800 ms. The presentation order of different types of stimuli was randomized. This task lasted 4 minutes and 30 seconds.

#### MRI data acquisition

After informed consent was obtained, an informal interview was administered to check each participant’s language background, hand dominance, and neuropathological and psychiatric history after informed consent was obtained. To be familiarized with the tasks, the participant practiced short versions of the experimental tasks outside of the scanner. In this practice session, new stimuli not part of the fMRI sessions were used. All the images were acquired using a 3T Siemens scanner at the University of Pittsburgh. Gradient-echo localizer images were acquired to determine the placement of the functional slices. For the functional imaging, a susceptibility weighted single-shot echo planar imaging (EPI) blood oxygenation level-dependent method was used. Functional images were interleaved from bottom to top in a whole brain EPI acquisition. The following scan parameters were used: TR = 2,000 ms, TE = 25 ms, flip angle = 79°, matrix size = 64 × 64, field of view = 205 mm, slice thickness = 3.2 mm, and number of slices = 38. These scanning parameters resulted in a 3.2 mm × 3.2 mm × 3.2 mm voxel size. At the end of the functional imaging session, a high resolution, T1 weighted 3D image was acquired (magnetization prepared rapid gradient echo, TR = 1,640 ms, TE = 2.48 ms, TI = 800 ms, flip angle = 8°, matrix size = 256 × 256, field of view = 249 mm, slice thickness = 0.8 mm, and number of slices = 256). The orientation of the 3D volume was identical to the functional slices.

#### Imaging data analysis

We analyzed the data using SPM8 (Statistical Parametric Mapping; http://www.fil.ion.ucl.ac.uk/spm). The functional images were corrected for differences in slice-acquisition time to the middle volume and were realigned to the last volume in the scanning session using affine transformations. No individual runs had greater than 4 mm maximum movement for any subject in the x-plane, y-plane or z-plane. Furthermore, no individual runs had more than 3° of maximum displacement in rotation for pitch, yaw, or roll. An analysis of variance (ANOVA) with task as an independent variable showed no significant main effects on any of the above six dependent variables, suggesting the writing task did not have a greater head movement than the passive viewing task. All statistical analyses were performed on movement-corrected images. Co-registered images were normalized to the Montreal Neurological Institute (MNI) average template (12 linear affine parameters for brain size and position, eight nonlinear iterations, and 2 × 2 × 2 nonlinear basis functions). Statistical analyses were performed on the smoothed data (9 mm Gaussian kernel). Data from each participant were entered into a general linear model (GLM) using a block analysis procedure for the passive viewing task and an event-related analysis procedure for the imagined writing task with a canonical HRF. Statistics were calculated with a high pass filter (128 s cutoff period). To test the language effect, we took into account that Chinese condition had twice the number of stimuli as English by assigning English data a doubled weighting. Parameter estimates from contrasts of the canonical HRF in single subject models were entered into random-effects analyses. All whole brain results are reported at *p* < 0.001, uncorrected at the voxel level with a cluster greater than 40 voxels and FDR corrected *p* < .05 at the cluster level.

#### Group analysis for English L1 participants

For the English L1 participants, t-tests assessed the task difference between passive viewing and imagined writing in each language and the language effect on each task. (These are all new comparisons, unreported in Cao et al, 2013, which reported the language comparison only in the passive viewing task.) We did not perform a full factorial ANOVA of language by task, because we were not interested in an interaction between language and task, which would be due to the different language proficiency of L1 and L2. Instead, we were interested in examining the common patterns of task differences in the two languages and the common patterns of language differences in the two tasks. Therefore, we did a series of conjunction analyses: 1) across both languages to reveal the language-independent difference between reading and writing; specifically, we calculated English (reading>writing) ∩ Chinese (reading>writing), and English (writing>reading) ∩ Chinese (writing>reading); 2) across the reading and writing tasks to observe task-independent language differences; specifically, we calculated reading (Chinese>English) ∩ writing (Chinese>English), and reading (English>Chinese) ∩ writing (English>Chinese); 3) conjunction between reading and writing within each language to observe the regions shared by the two tasks in each language; specifically, we calculated reading ∩ writing in Chinese and in English separately; 4) conjunction between the language effect and the task effect. Specifically, we calculated (Chinese> English regardless of task) ∩ (writing > reading regardless of language), and (English>Chinese regardless of task) ∩ (reading>writing regardless of language). For the fourth conjunction analysis, we calculated (Chinese> English regardless of task) and (English>Chinese regardless of task) separately for Chinese characters learned through the character-writing condition and those learned through the pinyin-writing condition. All contrasts included in the conjunction analyses were thresholded at *p* < .001uncorrected at the voxel level, and we reported only results from the conjunction analyses that survived at FDR corrected *p* < .05 of the cluster level.

In order to understand why there was overlap between the language effect (Chinese>English) and the task effect (writing>reading) (conjunction analysis #4) at the left MFG only for characters learned through character-writing but not for characters learned through pinyin-writing, we ran a ROI analysis at the left MFG (-50, 2, 28) which was the peak in the conjunction analysis of (Chinese> English regardless of task) ∩ (writing > reading regardless of language). This ROI analysis was done in the English L1 participants to demonstrate brain activation at this region in each type of stimuli (character-writing learned Chinese characters, pinyin-writing learned Chinese characters, English words) in each task (reading and writing). We defined a 6mm radius sphere centered at the peak, then we extracted beta values for each type of stimuli in each task. We then ran a paired-t test to directly compare brain activation during the reading task for Chinese characters learned through pinyin-writing with Chinese characters learned through character-writing. We also ran a paired-t test to compare brain activation for reading Chinese characters learned through pinyin-writing and reading English words, and another paired-t test to compare reading Chinese characters learned through character-writing and English words. These are the critical comparisons for examining how the writing experience influences brain activation of reading.

#### Group analysis for Chinese L1 participants

We did the same t-tests on the Chinese L1 participants to examine language effect (Chinese vs. English) and task effect (reading vs. writing) as we did with the English L1 participants. The main purpose of including the CHineseL1 participants was to exclude the possibility that language differences were due to difficulty associated with proficiency (L1 vs. L2) in our findings from the English L1 participants. Therefore, we did ROI analyses for Chinese L1 participants at regions where we had important findings related to language difference in the English L1 participants. The first ROI was the left MFG, which was the peak from the conjunction analysis between Chinese > English and writing > reading at (-50, 2, 28). The second and third ROs were the bilateral STG, which were the peaks in the conjunction analysis of reading (English>Chinese) ∩ writing (English>Chinese) for the English L1 participants at the left STG (-58, -66, 16) and the right STG (62,-56, 12). We defined a 6mm radius sphere centered at the peak of each ROI, and extracted beta values for Chinese reading, Chinese writing, English reading and English writing in our Chinese L1 participants. We then ran a 2 language (English, Chinese) by 2 task (reading, writing) repeated measure ANOVA for each ROI to find out whether that region shows the same language difference pattern in our Chinese L1 participants as in the English L1 participants. If so, it would then rule out the possibility of difficulty effect in our English L1 participants and suggest that this is a true language difference. Otherwise, it would suggest that these ROIs are sensitive to difficulty.

## Results

### Behavioral results

Behavioral results on the training and post-test were published in Cao 2013. The two learning conditions produced similar level of proficiency as measured in the meaning and pronunciation recall post-test. Both the passive viewing and the imagined writing task in the scanner did not require a behavioral response.

### Brain results

#### Task effects

**Fig 1:**
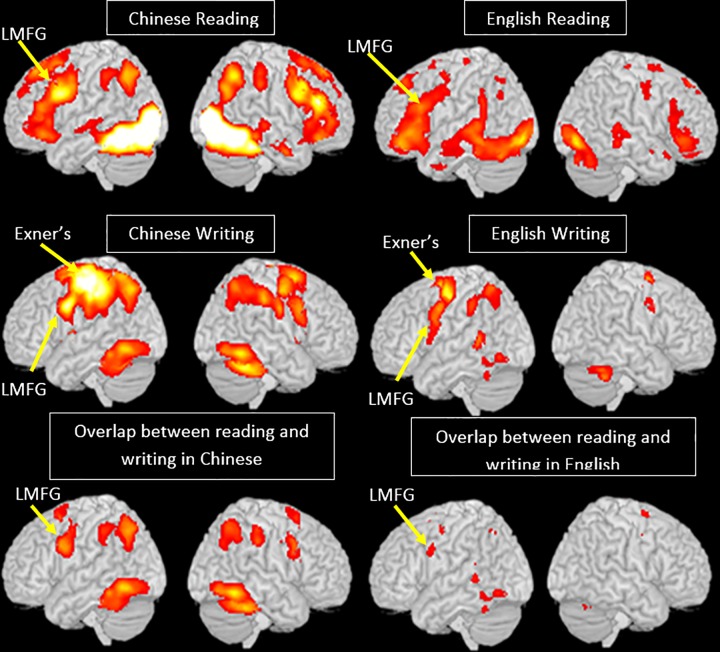
Brain activation for each task in each language. Chinese reading, English reading, Chinese writing, English writing, and the overlap between reading and writing in Chinese and English in the English L1 participants.

**Conjunction analysis #3 reading ∩ writing in Chinese; reading ∩ writing in English.** We found that the activation areas for reading and writing overlapped in the left temporo-occipital region, left middle frontal gyrus, and cingulate gyrus for both Chinese and English. (Fig 1, Table 1)

**Table 1:** Overlap between reading and writing for each language in English L1 participants.

Region	Overlap between reading and writing							
	Chinese				English			
	x	y	z	Z	x	y	z	Z
Left precuneus	-28	-66	40	7.77				
Left middle occipital gyrus	-44	-68	-8	7.74	-46	-68	-12	3.78
Right precuneus	30	-64	40	7.16				
Right inferior temoral gyrus	50	-62	-14	6.81				
Cingulate gyrus	-6	12	46	6.82	-2	8	56	3.40
Left middle frontal gyrus	-48	4	32	6.82	-48	4	32	3.76
Left insula	-30	18	6	5.28				
Right middle frontal gyrus	40	4	28	5.08				
Left middle temporal gyrus					-46	-44	4	3.91
Left fusiform gyrus	-50	-60	-16	6.63	-42	-52	-12	3.39
Left culmen					-42	-54	-28	3.47

**Fig 2:**
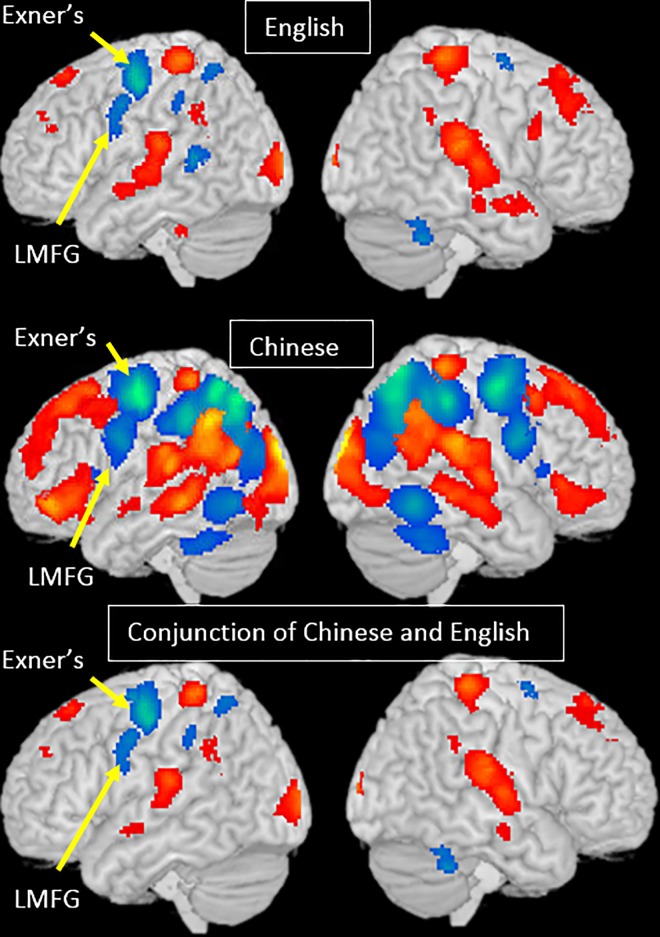
Task effect in each language, and the conjunction between the two languages. Brain activation for reading greater than writing is in red and writing greater than reading is in blue.

**Conjunction analysis #1 English (reading>writing) ∩ Chinese (reading>writing); English (writing>reading) ∩ Chinese (writing>reading).** We found that reading evoked greater activation than writing in bilateral STG, supramarginal gyri, postcentral gyri, cingulate cortex, insula, left cuneus and right SFG for both Chinese and English. For both Chinese and English, writing is associated with greater activation than reading in left premotor area, left MFG, IPL, SPL and right cerebellum (Table 2, Fig 2).

**Table 2:** Brain regions that are activated in the comparison between reading and writing for English, Chinese and both in English L1 participants.

Region	Reading>Writing											
	English				Chinese				Conjunction			
	x	Y	z	Z	x	y	z	Z	x	y	z	Z
Left cuneus	-12	-100	6	3.44	-8	-96	22	6.58	-12	-100	6	4.72
Left insula	-38	-26	16	4.53	-36	-26	20	4.87	-38	-26	16	4.53
Left Postcentral gyrus, BA 3	-18	-38	64	4.81	-18	-42	64	5.65	-16	-42	66	4.76
Left Anterior cingulate	-10	36	18	3.95	-12	32	48	4.69	-2	42	24	3.60
Right postcentral gyrus	22	-38	64	4.88	22	-38	64	5.08	22	-38	64	4.88
Right insula	36	-26	18	4.86	50	-30	22	5.37	38	-26	18	4.69
Right supramarginal gyrus	58	-44	32	3.41	64	-48	28	4.74	58	-44	32	3.41
Right superior frontal gyrus	22	34	38	3.77	22	38	42	3.39	22	36	42	3.33
Left supramarginal gyrus, BA 40	-64	-48	32	3.73	-62	-54	24	5.17	-64	-48	32	3.73
Left inferior parietal lobule, BA 40					-58	-54	40	5.95				
Left middle frontal gyrus					-48	44	-8	5.36				
Left superior temporal gyrus, BA 42					-62	-30	14	5.20				
Left superior temporal gyrus					-48	-58	22	5.19				
left middle temporal gyrus					-52	-36	-8	5.17				
left middle frontal gyrus					-44	18	46	3.82				
Left superior temporal gyrus, BA 21					-40	-6	-10	3.36				
Right cuneus, BA 19					20	-94	20	5.19				
Right inferior parietal lobule, BA 40					56	-56	40	4.93				
Right superior frontal gyrus, BA 8	6	36	56	4.04								
Right middle temporal gyrus	58	-2	-16	3.80								
Right inferior frontal gyrus	38	16	24	3.67								
Region	Writing>Reading											
	English				Chinese				Conjunction			
	x	Y	z	Z	x	y	z	Z	x	y	z	Z
Left middle frontal gyrus, BA 6	-24	-14	50	6.18	-26	-10	56	inf	-24	-14	50	6.18
Left middle frontal gyrus, BA 9	-58	-2	38	4.53	-48	0	28	6.28	-58	-2	38	4.53
Left precuneus, superior parietal lobule, BA 7	-30	-58	60	4.09	-30	-58	58	inf	-30	-58	60	4.09
Left inferior parietal lobule	-42	-38	42	3.54	-40	-40	40	7.67	-42	-38	42	3.54
Right culmen	46	-50	-36	4.78	44	-48	-34	5.11	46	-50	-36	4.72
Left inferior temporal gyrus					-46	-68	-8	6.18				
Left culmen					-34	-50	-32	4.87				
Right middle frontal gyrus					24	-6	54	inf				
Right inferior frontal gyrus					50	4	24	6.64				
Right superior/inferior parietal lobule					18	-64	62	inf				
Right inferior temporal gyrus, BA 37					52	-56	-12	6.80				
Left superior temporal gyrus	-36	-48	8	4.85								

For Chinese, reading also evoked more activation than writing in bilateral inferior parietal lobule, left STG, MTG, left inferior frontal gyrus and right cuneus, while writing evoked greater activation than reading in left MOG, right ITG, right IFG, MFG and right IPL/SPL. For English, reading also evoked more activation than writing in right IFG, right SFG and right MTG, while writing showed more activation than reading in left STG (Table 2, Fig 2). Regions that showed a significant task effect in only one language may or may not survive in an interaction test of task by language.

#### Language effects

**Fig 3:**
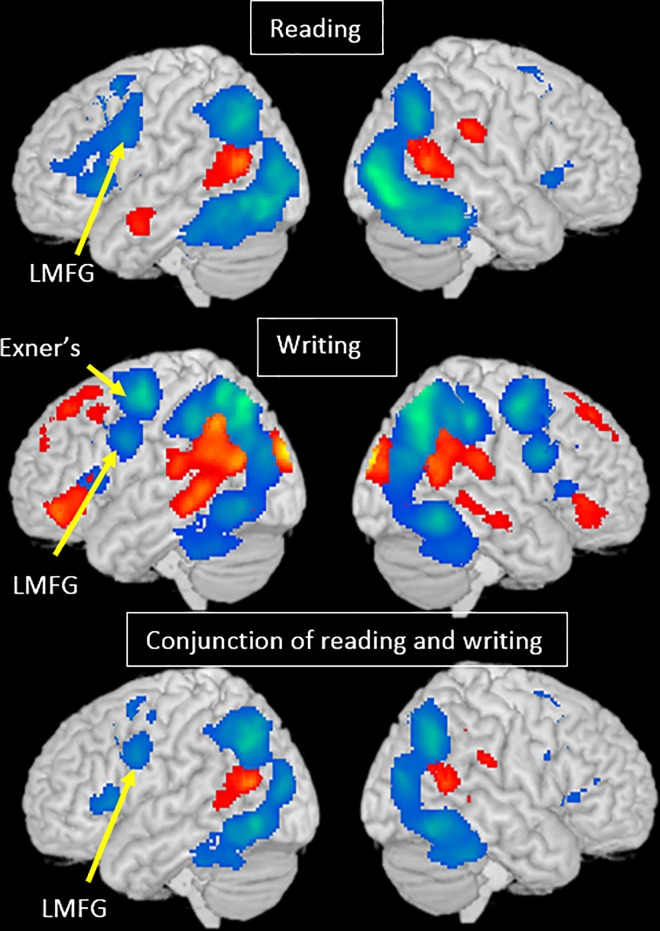
Language effect for each task, and the conjunction between the two tasks. Brain activation for English greater than Chinese is in red and Chinese greater than English is in blue.

**Conjunction analysis #2 reading (Chinese>English) ∩ writing (Chinese>English); reading (English>Chinese) ∩ writing (English>Chinese).** We found greater activation for Chinese than English in the bilateral occipital cortex, bilateral insula, bilateral precuneus, left MFG (BA 9), and cingulate cortex for both tasks. In contrast, we found greater activation for English than Chinese in the bilateral STG and right IPL for both tasks. (Table 3 and Fig 3)

**Table 3:** Brain activation for the language difference on the reading task, writing task and both in the English L1 participants.

Region	Chinese>English											
	Reading				Writing				Conjunction			
	x	y	z	Z	x	y	z	Z	x	y	z	Z
Right inferior occipital gyrus, BA 19	44	-80	-10	7.54	52	-56	-12	7.29	48	-64	-14	5.58
Left inferior occipital gyrus, BA 19	-42	-80	-8	6.51	-46	-68	-8	6.42	-42	-68	-8	5.97
Left middle frontal gyrus, BA 9	-48	4	32	4.66	-48	2	30	5.96	-48	4	32	4.66
Left insula	-30	18	2	4.76	-30	18	8	4.68	-30	18	4	4.56
Right insula	26	16	0	3.98	32	22	8	4.05	32	20	2	3.27
Left precuneus	-28	-66	40	6.22	-16	-70	52	inf	-28	-66	40	6.22
Right precuneus	30	-64	36	5.94	24	-66	48	inf	30	-64	36	5.94
Left cingulate gyrus	-6	14	46	5.70	-4	12	46	5.57	-6	12	46	5.56
Left middle occipital gyrus	-32	-88	10	5.80	-34	-82	22	6.20	-32	-86	14	5.14
Right superior occipital gyrus	34	-76	24	3.71	32	-74	24	6.10	38	-82	16	5.13
Left middle frontal gyrus, BA 6					-26	-8	56	7.78				
Right middle frontal gyrus, BA 6					24	-6	54	7.68				
Right middle frontal gyrus, BA 9					50	6	26	6.56				
Left culmen					-34	-50	-32	4.82				
Right culmen					36	-44	-30	4.71				
	English>Chinese											
	Reading				Writing				Conjunction			
	x	y	z	Z	x	y	z	Z	x	y	z	Z
Left superior temporal gyrus	-58	-66	16	4.95	-54	-44	2	4.77	-58	-66	16	4.95
Right superior temporal gyrus, BA 22	62	-58	10	4.68	66	-38	-2	3.58	62	-56	12	4.39
Right inferior parietal lobule	56	-32	28	4.13					54	-32	26	3.70
Left inferior temporal gyrus, BA 21	-62	-6	-22	3.91								
Left cuneus, BA 19					-8	-96	22	6.06				
Right cuneus					20	-92	20	4.55				
Left IPL, BA 40					-58	-54	40	5.23				
Right STG					60	-62	22	4.90				
Left inferior frontal gyrus					-48	42	-10	4.73				
Right inferior frontal gyrus					54	32	-2	3.90				
Left dorsal middle frontal gyrus					-38	18	46	3.40				

In addition, for the writing task, there was also greater activation in bilateral MFG and bilateral cerebellum for Chinese than for English, while there was greater activation for English than Chinese in bilateral cuneus, bilateral STG/IPL, bilateral IFG and left dorsal MFG. (Table 3 and Fig 3)

#### Overlap between the language effect and the task effect

**Fig 4:**
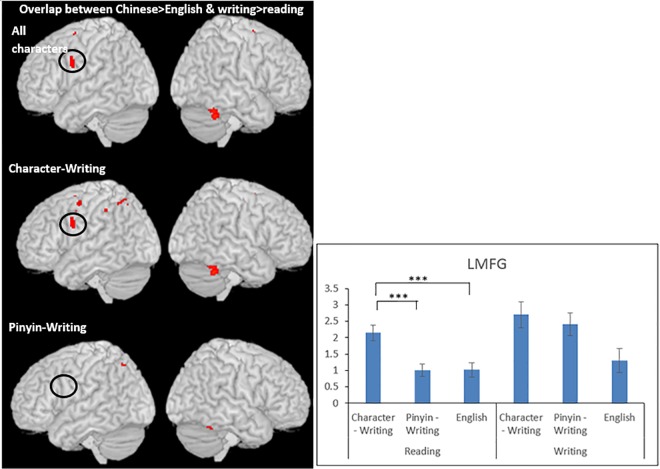
Overlap between Chinese greater than English and writing greater than reading. The results are presented for all Chinese characters, Chinese characters learned in the character-writing condition and characters learned in the pinyin-writing condition for the English L1 participants. Bar graph shows the brain activation at the left middle frontal gyrus at (-50, 2, 28) in each task for characters learned in either character-writing or pinyin-writing and for English words. *** indicates significance at p < .001. Error bars indicate standard errors.

**Conjunction analysis #4 (Chinese> English regardless of task) ∩ (writing > reading regardless of language); (English>Chinese regardless of task) ∩ (reading>writing regardless of language).** The left middle frontal gyrus showed greater activation in Chinese than English for both the reading and the writing task; this same region showed greater activation for writing than reading in both Chinese and English. Cingulate cortex and right cerebellum were also significant in the conjunction between the language effect and the task effect. See Table 4 and Fig 4. In order to examine whether the LMFG is related to the writing experience, we calculated the overlap separately for characters learned in the character-writing condition and characters learned in the pinyin-writing condition. We found that only for characters that were learned in the character-writing condition, there was overlap between Chinese>English and writing>reading in the left MFG. For characters learned in the pinyin-writing condition, there was no overlap between Chinese>English and writing>reading. The ROI analysis at (-50,2, 28) in paired-t tests revealed significant greater activation for reading Chinese characters learned through character-writing than those learned through pinyin-writing (t(16) = 9.423, P < .001) (Bonferroni correction P < .016 = .05/3), and than reading English words (t(16) = 5.245, P < .001). However, there was no difference between reading Chinese characters learned through pinyin-writing and English words (t(16) = 0.227, P = 0.823). This explains why there was overlap between writing>reading ∩ Chinese>English only for Chinese characters that were learned through character-writing, not for those learned through pinyin-writing. This was because the left MFG was activated in Chinese>English only for Chinese characters learned through character-writing but not for pinyin-writing. We found no overlap between English>Chinese and reading>writing in the conjunction analysis.

**Table 4:** Overlap between the language effect and task effect for all characters, characters learned in the character-writing condition and characters learned in the pinyin-writing condition for English L1 participants.

All characters				
	Overlap between Chinese>English and writing>reading			
	x	y	z	Z
Right culmen	44	-50	-32	4.00
Right culmen	32	-52	-28	3.86
Left middle frontal gyrus	-50	2	28	3.62
Cingulate	0	2	58	3.61
**Character-writing learned**				
Cingulate	0	2	58	3.61
Left middle frontal gyrus	-50	2	28	3.62
Right culmen	32	-52	-28	3.86
**Pinyin-writing learned**				
	—			

#### Chinese L1 participants

**Fig 5:**
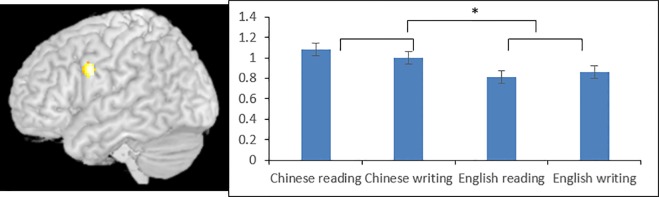
ROI analysis at the left middle frontal gyrus in the Chinese L1 group. Chinese L1 participants also showed greater activation for Chinese than for English at this ROI centered at (-50, 2, 28). * indicates significance at p < .05. Error bars indicate standard errors.

**Fig 6:**
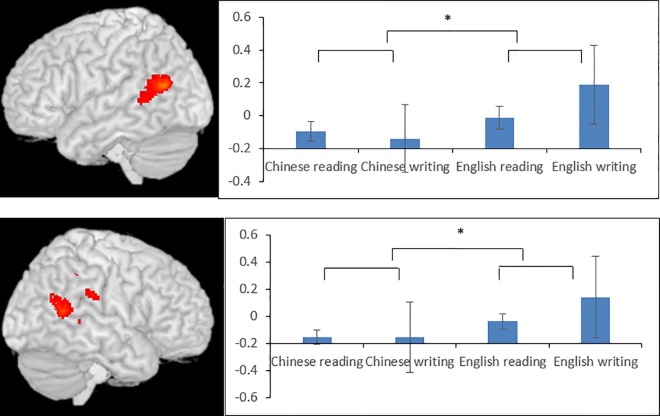
ROI analysis at the bilateral superior temporal gyri in the Chinese L1 group. The center is at (-58, -66, 16) for the left STG and at (62, -56, 12) for the right STG. Chinese L1 participants showed greater activation for English than for Chinese at these two regions. * indicates significance at p < .1. Error bars indicate standard errors.

The whole brain analysis on language and task effects in the Chinese L1 participants revealed results very similar to the English L1 participants. To avoid repetition, we focus only on the ROI analyses on the Chinese L1 participants, which are shown in Fig 5 for the LMFG and Fig 6 for the STG. As can be seen in Fig 5, The main effect of language was significant at LMFG F(1,16) = 4.615, P = .047, with Chinese characters associated with greater activation than English words (Table 2 and Fig 3). The main effect of task F(1,16) = 0.003, P = .961 and the interaction between language and task F(1,16) = 0.381, P = .546 were not significant.

The ROIs for the STG (Fig 6) were defined as the peaks of the conjunction between Reading (English>Chinese) and writing (English>Chinese) (Table 2, Fig 3). Using a very liberal threshold of p < .1, we found an effect of language (of borderline significance) with greater activation in English than in Chinese at the left STG (F(1,16) = 4.212, P = .057) and right STG (F(1,16) = 3.586, P = .077). The main effects of task for both left and right STG were not significant, both Fs < 1, and task did not interact with language at either left STG (F < 1) or right STG (F (1,16) = 1.47, P = .242).

## Discussion

### Left MFG

The most important findings in this study concern the functionality of the left MFG in reading and writing across Chinese and English. A major conclusion from these results is that the LMFG is activated in both tasks and both languages, but more strongly for writing than reading, and more strongly for Chinese than English. The conjunction analyses imply that cortical areas at or near the LMFG support a motor component of writing that functions during reading when writing has been a part of the reading experience. Thus, during reading, the LMFG is more activated for Chinese than for English, because the acquisition of Chinese literacy involves more hand writing than does English literacy. The finding that the LMFG is more activated during the reading of characters that had been learned through writing, compared with those learned through pinyin, provides direct evidence for our hypothesis. We briefly summarize the key findings that support these conclusions.

First, the LMFG was activated in both reading and writing with a peak of their overlap at (-48, 4, 32). Previous studies that have included both reading and writing tasks for the same individuals have had similar findings at (-42,12,30) in Purcell, 2011 [26], at (-48,6,20) in Rapp, Lipka 2011 [52], at (-42,-2,27) in Rapp 2011 [27], at (-53, -6, 41) in James 2006 [43], and at (-53,4,40) in Longcamp 2003 [41]; except for Purcell et al (2011), these studies examined the overlap between reading and writing without directly comparing them. We found that writing produced more activation than reading at a point more lateral and slightly more superior (-58, -2, 38) compared with the peak of overlapped activation of reading and writing (-48, 4, 32), and also compared to the area reported by Purcell et al (2011) for the peak of greater activation for writing than reading (-48, 6, 28). However, when the threshold was lowered, the overlap region between reading and writing was also greater for writing than reading. Thus, although this region is involved in both reading and writing, it is more involved in writing than in reading.

Adding the language comparisons produces a series of important interlocking results. First, Chinese reading and writing showed greater activation than English reading and writing at (-48, 4, 32). Second, this language difference (Chinese>English) overlapped with the task difference (writing>reading) at (-50, 2, 28). Third, this region (-50, 2, 28) showed greater activation during the viewing of characters learned through character-writing than the viewing of characters learned through pinyin-writing, even though participants reached the same level of proficiency on these two types of characters by the end of training, as tested in a meaning and sound recall post-test. The only difference between the two conditions was that for characters in the pinyin-writing learning condition, participants did not have an experience of writing the characters. Fourth, at this same region (-50, 2, 28), Chinese L1 subjects also showed greater activation for Chinese reading and writing than English reading and writing, suggesting that difficulty alone cannot drive the language effect in this region. This set of results points to the general conclusion we suggested above: Cortical areas at or near the LMFG function to support a visuo-motor component of writing that activates during reading when writing has been experienced as part of learning to read. We now consider how these conclusions comport with previous research and interpretations of LMFG functions.

Some previous studies have shown that when participants viewed visual words/letters that they had been previously written, brain activation could be observed in the writing-related sensori-motor network, including the left MFG [28,40,41,53–55]. Interestingly, Nakamura et al., 2012 found that French and Chinese readers showed the same pattern of priming effect in the left MFG (-42, 6, 20) when they viewed the forward moving trajectory of handwritten words. Our findings suggest a cross-linguistic difference in the involvement of the left MFG during reading, presumably due to the different degree of writing experience during learning. The finding of reading-writing co-activation is consistent with the principle of perception of dynamic information in static handwritten forms [56,57].

To add to this perception-action function is evidence that this region is involved in visuo-orthographic processing. For example, damage to this region causes acquired dysgraphia in acute stroke [58], suggesting its role in orthographic long term memory. Another study found that the pattern of sensitivity to orthographic frequency at the left MFG (-49, 1, 33) was the same as at the left fusiform gyrus [27]. These two functions, writing and visual-orthographic processing, are inter-connected. Both visuo-orthographic retrieval and writing-related motor information are required more in writing than reading, and more in Chinese than in English. In order to produce orthography, detailed information of a graphic form has to be retrieved from long-term memory, whereas reading demands only that the presented form can be recognized or distinguished from other forms. Furthermore, Chinese places greater visual memory demands on reading than does alphabetic writing, because it requires discrimination among thousands of characters. Writing-specific motor information can support orthographic processing against the high demands of written Chinese. Native Chinese speakers learn to read by repeatedly copying and writing characters in specific sequences of strokes, providing this support and leading to the activation, by reading, of writing-related brain areas. Our current experimental design cannot disentangle the two hypotheses about the function of the left MFG; however, evidence that this region has a specific motor component comes from a study [29] that directly compared left handed writers and right handed writers while they viewed letters. The results were greater activation in the left MFG for right handed writers and greater activation in the right MFG for left handed writers, with symmetrical clusters at (-51, -2, 41) and (51,-2, 39). This suggests that this region is related to specific writing experience in a situation of low orthographic demands. These clusters at MFG are proximal to our writing region (-58, 2, 38). However, the tight coupling of the visuo-orthographic and motor processes makes their clear functional separation difficult.

It is important to recognize that the left middle frontal gyrus may be important in a wide variety of functions involved in many cognitive domains. Its role in reading and writing, however, is greater for Chinese than English and greater for writing than reading, and any explanation of how its functions support literacy behaviors has to account for these facts. Our results show that one viable explanation is that, within this region, writing-specific visuo-motor information is functional. Further evidence especially from lesion studies would be helpful to understand its functions in Chinese and English literacy.

### Writing specific region

Two previous meta-analyses studies of writing studies have argued that the left MFG is related to the central linguistic processing of writing and reading, whereas Exner’s area is related to writing-related peripheral processes [21,22]. This is because the LMFG was found to be shared with reading, whereas Exner’s area is writing-specific [27,41,52]. Our findings are consistent with this central versus peripheral distinction between the left MFG and Exner’s area, with the additional implication that its “central linguistic” role includes a motor preparation component. The left MFG has a role in processing the visuo-orthographic and gestural information intrinsic to writing, making it available to writing related motor planning in Exner’s area.

We found Exner’s area (-24, -14, 50) was purely writing motor related, activated only during the imagined writing task. We found that it was more involved in Chinese writing than in English writing, and that reading did not activate the Exner’s area in either language. Previous studies have also found that viewing letters/reading activated the left MFG but not the Exner’s area [29,41][42], even though the authors did not explicitly and clearly discuss the different functions of the left MFG and Exner’s area in reading and writing. Longcamp, 2003 found that reading and writing overlap at the left MFG (-53, 4, 40), but only writing showed activation in the more medial Exner’s area (-28, -1, 65), although it was not directly tested whether the Exner’s area is more activated in writing than reading. Another study found viewing letters activated the left MFG (-44, 3, 29) but not Exner’s area [29]. Exner’s area has traditionally been associated with a role in the generation of motor commands for handwritten letters [23,25,59]. It has been repeatedly found to be more activated for writing than reading (-22,-4, 50) in Purcell, 2011, and (-24,-16, 68) in [60]. This region is involved in both handwriting and typewriting [26,60], suggesting its importance for written language production rather than specific motor commands per se, and is sensitive to word-length in spelling [27], which is consistent with our finding that it is more involved in Chinese writing than in English writing due to more complex written output. However recent work on stroke patients suggests that it may also have a role as a graphic buffer, a working memory component of the spelling system that temporarily holds the sequence of graphemes during production of letter names for oral spelling or letter shapes for written spelling [61].

### Temporo-occipital areas

Our results for posterior left hemisphere areas are consistent with the many studies showing the activation of the left mid-fusiform gyrus (the “visual word form area”) by orthographic input [18,62–64]. Other studies have shown that this area is activated not just for reading, but also for writing [26,27,52,63,65,66]. Consistent with these findings, we found an overlap between reading and writing in the left mid-FG that peaked at (-42, -52, -12) near to previously reported areas–(-42, -48, -13) in [52], (-44,-52,-14) in [26], and (-43, -54,-7) in [27]. Our study adds the result that sharing of the VWFA for reading and writing occurs in both L1 and L2. In a slightly more posterior region that peaked at (-46,-68,-8), we found greater activation for writing than reading only in Chinese. This replicates a result of Purcell in which this area (-48, -64, -6) was found to be more involved in writing than in reading [26]. This more lateral and superior region within VWFA is involved in multi-modal visual/auditory processing, whereas a more medial part of the VWFA is associated with unimodal visual word processing [67]. This region has also been found to be related to single letter working memory in adults and to scores on a standardized spelling test in children [68]. Other studies have found that this region showed greater activation for viewing letters that have been learned by handwriting than by typing or visual only in adults with a peak at (-43, -66, -12] [69], as well as in young children [42,55,70]. This may suggest that this more posterior and superior aspect of the fusiform gyrus is specifically related to spelling; however, it may also be because writing tasks are more demanding on orthographic processing. In contrast to the attempt to find specific orthographic representation for reading and spelling in the fusiform gyrus [71], we found that the shared regions of reading and writing were also more activated in writing than reading when the threshold for the writing > reading contrast was lowered to .005. The fusiform gyrus is involved in both reading and writing for orthographic processing, but its superior and posterior aspect is more involved in writing than reading due to the greater demands on orthographic representations in written word production.

At the left middle occipital gyrus, we found greater activation in reading than writing with a peak at (-12, -100, 6) for both Chinese and English. This region is anatomically connected to the inferior frontal gyrus and temporal pole via the mid-posterior fusiform gyrus through the inferior frontal occipital fasciculus [72]. This region is an important pathway in naming and visual language processing, more specifically in its semantic aspect [73]. It may show more activation in reading than in writing in our study because of the longer duration of visual stimuli (800ms) in the reading task than the writing task (200 ms).

At the bilateral temporo-occipital regions, we found greater activation in Chinese than in English for both reading and writing. This may be due to the greater complexity of the Chinese visual form than English visual form, and it converges with previous comparisons of Chinese and English in bilateral temporo-occipital cortex [2,3].

### Temporo-parietal regions

We found greater activation in reading than in writing in bilateral temporo-parietal regions in both Chinese and English. We also found that, at a slightly different location, English showed greater activation than Chinese in bilateral STG regardless of task. These regions have been found to be related to phonological representation and orthography-phonology-conversion [63] with stronger phonological activation in English L1 reading than Chinese L1 reading in the superior temporal gyrus [7]. Our results are consistent with this language difference in STG found in previous studies and add that it occurs for writing as well as reading. Thus, our findings again suggest that phonological regions are more involved in reading than in writing regardless of language and that the phonological regions are more involved in English than in Chinese in both reading and writing, reflecting the English’s intimate sublexical relationships between orthography and phonology. Our ROI analysis for the Chinese L1 participants at the language effect regions in bilateral STG showed the same pattern: that this region is more involved in English than Chinese regardless of task, even though the language difference was only marginally significant at both the left STG and right STG. Both English L1 and Chinese L1 participants showed greater activation in these regions for English than Chinese, suggesting that these phonological regions are indeed more involved in English than in Chinese due to the features of the written language rather than language proficiency.

## Conclusion

Our most important result concerns the function of the left middle frontal gyrus. Our comparisons across reading and writing tasks and across Chinese and English, combined with a learning study that manipulated character writing as part of learning and comparisons of English learners of Chinese with Chinese native speakers, allow a clearer picture of the function of the LMFG during reading and writing. First, we replicated previous findings in alphabetic languages that reading activates regions that are involved in writing. Second, by directly comparing Chinese and English, we found that this reading-writing co-activation is greater in Chinese than in English. Third, we explained why this is the case by comparing characters learned through writing and those learned through pinyin. The LMFG is indeed more involved for Chinese reading than English reading, because it functions in support of the orthographic-motoric aspects of writing. Because Chinese literacy requires much more writing than alphabetic literacy, Chinese character reading entails knowledge of character writing. This knowledge, we suggest, consists of the sequence of strokes that is defined for each character and acquired by learners. The functioning of this knowledge is reflected in the greater involvement of the LMFG in writing compared with reading, with Chinese compared with English, and with viewing characters that were learned through character writing compared with those learned without character writing. Last, we replicated our key findings in a group of Chinese L1 speakers to avoid the possibility that our findings in English L1 speakers are due to language proficiency effect. From a cross-linguistic perspective, our study, for the first time, demonstrated that learning experience modulates brain function.
